# Older people living alone (OPLA) – non-kin-carers’ support towards the end of life: qualitative longitudinal study protocol

**DOI:** 10.1186/s12877-019-1243-7

**Published:** 2019-08-13

**Authors:** Sabine Pleschberger, Elisabeth Reitinger, Birgit Trukeschitz, Paulina Wosko

**Affiliations:** 1 Gesundheit Österreich GmbH (GÖG, Austrian Public Health Institute), Vienna, Austria; 2 Department of Nursing Science, University of Vienna, Vienna, Austria; 3 Research Institute for Economics of Aging, Vienna University of Economics and Business, Vienna, Austria

**Keywords:** Older people, Living alone, Non-kin-carers, End-of-life care, Gender issues

## Abstract

**Background:**

A growing number of older people, mainly women, live in single households. They represent a vulnerable group as staying at home may turn out challenging when care needs increase, particularly at the end of life. Non-kin-carers can play an essential role in supporting individuals’ preferences to stay at home. In research little attention has been paid to non-kin-carers, such as friends and neighbors, yet. Thus, the Older People Living Alone (OPLA) study will evaluate whether non-kin support is robust enough to enable care dependent people to stay at home even at the end of life. This paper aims to introduce the research protocol.

**Methods:**

We plan to apply a qualitative longitudinal study to better understand how older people living alone and their non-kin-carers manage to face the challenges with increased care needs towards the end-of-life. We will conduct serial interviews with the older persons living alone and their non-kin-carers. A total of 20–25 complete data sets and up to 200 personal interviews were planned. These will be complemented by regular telephone contacts. All interviews will be analysed following the grounded theory approach and strategies for reconstructing case trajectories, supported by MAXQDA software. In the course of the study, inter- and transdisciplinary workshops shall assure quality and support knowledge transfer.

**Discussion:**

This study protocol aims to guide research in a field that is difficult to approach, with regard to its topic, methodology and the interdisciplinary approach. As this study introduces longitudinal qualitative research methodology in the field of home care in Austria, a deeper understanding of (end-of-life-) care trajectories will be enhanced, which is of major relevance for future care planning. With investment in additional reflexivity and communication procedures innovative results and robust knowledge are expected outcomes.

## Background

Older people, who live alone, form an increasing group in many countries. In Austria, 51,4% of all households of people aged older than 65 years were single-households in 2018 [1]. This phenomenon is quite common in Europe, where more than a third of all people at the age group of 65+ years (31.6% on average) live alone [2]. This amount is increasing by age and more women are affected than men as data from Austria show: 59% of all women aged 80 and older lived alone in 2018, while this was the case for only 24.4% of men of the same age group [3]. As an enormous increase of single-households in the group of 65+ is expected for the next decades [4], the issue of older people, who live alone, will be of major relevance for many societal systems, particularly for the health and social care systems.

Old age usually comes along with an increased risk of health problems that often also imply comprehensive care needs that need to be met. In the last decades, health and social care systems have been developed and redesigned to support people’s preferences for staying at home as long as possible [5]. Older people living alone share these preferences [6] but face enormous challenges due to shortcomings of the wider health and care systems, e.g. the absence of care concepts for this group which sufficiently consider the role of informal carers [7, 8]. However, informal caregivers are not a homogenous group [9]. Informal caregiving is generally understood as family caregiving, whereas little regard has been paid to non-kin-carers, such as friends and neighbors [10].

It is generally understood that older people prefer to ‘age in place’ rather than move to care homes and this notion is reflected in government policies and services that aim to allow older people aging in place [11, 12]. Certainly, informal care does play a vital role in these arrangements. For older people who live alone these are particularly shaped by their neighborhood and the engagement of non-kin-carers [13, 14]. Usually, these support arrangements do not exist between complete strangers, although the neighborhood relationships are often rather superficial before [15]. The helping arrangements usually develop from so called ‘small beginnings’ for instance spontaneous support with shopping, transport to hospital or practical repairs [16]. As Van Dijk et al. (2013) put it, ‘From nothing it became a lot’ [14]. Especially the evolvement of ‘bonding relationships’ is of interest with regard to staying at home until at last [13].

Research suggests considering different forms of non-kin-carers, such as neighbors or friends, who differ with regard to motivation, assumption of tasks and intensity of involvement [16, 17]. In a Canadian study, neighbors as informal carers turned out to be much younger than caring friends. On average, however, friends invested more hours per week supporting their friend in need [18]. An exploratory study commissioned by the Austrian Ministry of Social Affairs on older people living alone with increasing care needs shed light on the substantial involvement of non-kin carers [10]. It is important to determine whether this kind of support can be robust enough to enable staying at home even at the end of life.

Place of care and place of death are important for the quality of end-of-life care. Against this background, ‘dying with dignity’, which for most people living alone is synonymous with staying at home for as long as possible, is one of the most important “existential considerations” [6]. Though older people living alone possess various strategies to remain independent, they turn out to be vulnerable, due to their difficult living situations, limited resources or lack of support [19]. Hanratty et al. (2013) estimated chances of older people who lived alone to stay at home for as long as possible as very pessimistic, but the prospect of moving into a nursing home was seen as a real source of anxiety by them [20]. Further studies confirm that it is mainly a lot of concerns which make older people change their minds and shift their preferences from home to inpatient settings in the course of their last years and months [21, 22].

Literature does not provide a cohesive picture of non-kin involvement in end-of-life care but rather snapshots as illustrated in a scoping review [10]: Burns et al. (2011) found that friends tended to involve specialist palliative care services earlier and more often than family members did [17]. Furthermore, the need for Advance Care Planning might be more evident in case of older people living alone than when family carers are in the same home [23] . As this is not common practice, the person’s wish to die at home can easily be jeopardized [24].

Finally, gender represents and is represented within the societal power structures. The gendered system leads to a gendered division of (paid and unpaid) labor, and with this study we will shed light on both areas, as care work is mainly defined as “female” work [25].

In terms of family caregiving studies have highlighted the gendered nature of caregiving, indicating that women have been positioned as caregivers by health care providers as well as by family members [26, 27] . While men provided care mostly to their spouses, women took care for a broader range of people including partners, siblings, parents [28]. Most of the research on gender relations in hospice palliative home care has focused on sex and gender differences [29]. Limited exploration of how and why gendered processes are enacted in the various contexts and setting has been done yet. In order to change practice it is vital to better understand the “doing gender” in care for older people [30–32].

This paper presents the design of the research project on “Older People Living Alone (OPLA)”. The research project puts a focus on a so far neglected though increasingly relevant group of society, which is older people living alone with long-term conditions who receive support by non-kin-carers. The aims of the project are to [1] better understand the challenges that older people living alone face in light of increasing care needs towards the end of life, [2] analyse the potential of non-kin-carers in care arrangements of older people living alone and identify gender specific patterns therein, [3] work out characteristics of different arrangements which support older people’s preferences for staying at home including access to palliative care, [4] identify areas of improvement in the Austrian long-term care system with regard to supporting older people living alone including end-of-life care.

## Methods

In order to get an in-depth understanding of the challenges of older people living alone and the contribution of their non-kin-carers longitudinal case studies shall be generated through serial interviews [33, 34]. With this prospective longitudinal study design, we would like to overcome some of the shortcomings we usually face in end-of-life research:

- We know from research in the field of family caregiving that the burdens of care emerge from the duration of the care relationship and its progressive character as well as adaption processes have to be considered [35]. To better capture the dynamics in the relationships and care arrangements between informal carers and older persons it is essential to interview them more than only once and prospectively [36, 37].
- A longitudinal approach allows a better understanding of older people’s needs and preferences which might change towards the end of life [38, 39].
- Caregivers’ experiences with end-of-life care and their narratives differ when collected in retrospect, as mourning and grief and/or relief of burden influence perspectives [40, 41]. Therefore, a prospective design will reveal more robust information concerning support needs throughout the process of care including time of bereavement.

### Sampling and recruitment

The main target population of this study includes people living alone in a progressive state of illness or frailty being supported by an informal non-kin-carer, regardless of the intensity or character of support. As this applies to all qualitative studies, sampling does not aim at representativity [42]. Instead, the sampling aims to cover abroad variety of older people living alone at the end of life and their non-kin-carers in Austria.

Older people living alone and their non-kin carers are not easy to approach [24]. Therefore, various strategies will be applied, in order to avoid sampling bias and cover a broad variety of care arrangements (see Table 1).

**Table 1:** Access to the field

Gatekeeper	Aiming to recruit …
Home care services	… older people living alone without a specific disease
Specialist palliative care services	… older people living alone with a terminal illness
General Practitioners	… care arrangements independent of involvement of professional care services (via older people living alone as well as non-kin-carers who are patients of GP)

Recruitment will take place in four Austrian regions, Styria, Upper Austria, Lower Austria and Vienna in order to cover rural areas (Styria, Upper and Lower Austria) as well as urban areas (Graz in Styria and Vienna). The regions involved cover about 58% of the Austrian federal territory and 71% of the Austrian population.

Gatekeepers should inform the target group (care dependent people being looked after by a neighbor or friend) about the study and hand out an information sheet to potential participants. Upon agreement by the latter, gatekeepers will pass on contact details to the research team, which will get in touch with participants, explain the research aims and ask for consent.

#### Sampling criteria

A qualitative sampling strategy will be applied in order to cover some basic characteristics, which are relevant for this study, such as a) type of relationship to informal carer, b) gender and c) progression of disease [43].

- We are aiming to differentiate the group of non-kin-carers in our sample to work out the specific characteristics and challenges with regard to relationships: Neighbors and friends as non-kin-carers shall be part of the sample quite evenly.
- We consider gender a relevant criterion for sampling as we are aiming to include various combinations with regard to patients and carers. Considering the groups of interest here an overrepresentation of females seems to be inevitable – especially on the side of older people living alone. In order to contrast gendered phenomena like involvement in physical aspects of care, we look out to include about 8–10 male non-kin-carers and 6–8 older men living alone.
- Older people living alone should be included in a progressive state of their disease and/or stage of frailty in order to raise the probability to represent end-of-life issues in the trajectories. The only exclusion criterion is the inability to participate, for example, because of dementia, an immediately life threatening illness or a situation of crisis.

As we are aiming to include the end-of-life phase in the process observed it seems essential to consider progression of disease or frailty as a criterion for inclusion. Keeping in mind that this is a difficult endeavor, we will try to approach this by suggesting health professionals to reflect the “surprise question” when screening their records to identify potential participants: “Would you be surprised if this person were to die within the next year?” This indicator has proven useful in a similar study [39].

#### Timing and number of interviews

The aim is to capture change as it happens, and to respond to the individual dynamics in the care arrangements towards the end of life. For this reason, this study is designed along serial interviews with flexible intervals, as suggested by Carduff et al. (2015): After a baseline interview, we plan to conduct three personal interviews with the older person and the nominated non-kin-carer at least every 6 months over a period of 18 months. Telephone contacts with participants in between (about every 6 weeks) should ensure contact and help identify “critical situations” [44].

In case a patient dies within this period a retrospective interview (RI) will be performed with his/her main informal caregiver, about 3–5 months after the patient’s death. This extends the total period of data collection to up to approximately 20 months per case.

Pinnock et al. (2011) suggest that a total of 16–20 complete data-sets are necessary in order to reach sufficient data for profound analysis [39]. As we are aiming to contrast within our sample relating to selected criteria (gender issues as well as different types of non-kin-carers) we exceed this number to 20–25 complete data sets. The latter consist of those cases where we manage to conduct interviews at least at two points of time (longitudinal), as well as collecting data from the older person living alone and a non-kin-carer at each instant (two perspectives).

In longitudinal studies, attrition has to be considered. A certain drop out within the period of 18 months has to be expected either due to rapid progression and “hastened death” or other causes (withdrawal, non-ability to be interviewed due to worsening condition) [34]. However, most of these incidents are unavoidable in this group of interest, so we work with the construct of a minimum data set (see above). Furthermore, we engage in building sustainable relationships with the participants throughout the process. Regular telephone contacts will also contribute to reduce attrition [44]. Hence, we are planning to start with recruitment of about 30 cases at the onset, to gain a total of 20–25 complete data sets in order to avoid oversampling [33]. Apart from the length of the trajectories additional variability evolves from interviews which will be conducted jointly, as well as additional personal interviews due to acute crisis where necessary. The older people and their non-kin-carers will be interviewed separately, however, if they want to do one or more joint interviews we would allow this, too [34, 39].

In light of this, it is difficult to calculate an exact number of interviews planned. Therefore, we work with approximate numbers. We estimate an average value of about 180 interviews to be conducted within the study period. This number considers a minimum of 80 interviews with older people living alone and/or non-kin carers up to a maximum of 225 interviews as presented in Table 2.

**Table 2:** Number of interviews

Number of interviews per data set/case						
Time	T0	T1	T2	T3	RI^b^	Sum
Min	*1/1* ^*a*^	*1/1*			†*/0*	4
Max	*1/1*	*1/1*	*1/1*	*1/1*	†*/1*	9
	Number of interviews (Range):					4–9
Number of data sets/cases						
Time	T0	T1	T2	T3	RI	Sum
	*30*	*… estimated attrition over time …*				20–25
Number of interviews in total						
Min	*20 data sets á 4 interviews*					80
Max	*25 data sets á 9 interviews*					225
Estimated number of interviews (Calculated range: 80–225)						~ 180

Notes: ^a^(Older person/Non-kin-carer), ^b^(*RI* Retrospective Interview)

### Data collection

To generate the data-sets for the case studies serial interviews with the older person living alone and/or her non-kin-carers will be conducted. We would like to collect the two perspectives separately, however, if preferred, we would also interview simultaneously [34, 39]. To build up a trustful and sustainable relationship, which is crucial for longitudinal studies, all interviews within a trajectory shall be conducted by the same interviewer [45]. We apply narrative interviews as narratives have proven to produce the most suitable and saturated data to present perspectives of people on end-of-life issues [45, 46].

The initial interviews serve two goals. On the one hand, they aim to build a trustful relationship between researcher and participant [47]. This will be crucial in order to get high quality data and to motivate people for serial interviews [33]. Secondly, it is necessary to include information about the biography and current situation of the older people as well as their informal carers in order to make sense of the data. An interview guide will be prepared and piloted before starting the first baseline collection of data. It serves to capture the range and depth of the subject’s experiences, while being sufficiently flexible to enable the interviewer to respond to individual concerns. Interviews with non-kin-carers will aim to explore motivation for their commitment as well as limits or prior experiences with caring.

The follow-up interviews will be conducted by the same researcher as the initial interviews and will start with open stimuli to allow the participants to provide an update of their situation including key events in the foregoing weeks. Some issues will be checked regularly as integrated in a follow-up-interview guide, e.g. ideas about the future or issues of relationship between non-kin-carers and persons in need of care. The interview guide for the follow-up interviews will also consist of an individual part depending on the situation and dynamics of each case. This latter part will be informed by the telephone conversations between the personal interviews.

The telephone-interviews will consist of one or two questions aiming to recall the study and collect contextual information to the current situation of the care arrangement. The calls shall not be recorded to keep them quite informal and to build trust. However, the researcher will take notes right after the telephone call and these notes will be part of the data pool for analysis as well as useful information for the subsequent personal interviews.

In case of death of an interviewee, we will conduct a retrospective interview with the non-kin-carer. The aim of this interview is to focus on the last phase of life and specific conditions and challenges in the last days, as well as after death. Issues of bereavement in non-kin-carers will be part of these interviews, too. Timing of these retrospective interviews will depend on the non-kin-carer. Interviews with bereaved family members usually are conducted 3–5 months after death of the person [40], but an individual variation according to the closeness of the relationship as well as personal characteristics of the non-kin-carers have to be considered in this study.

All personal interviews will be voice-recorded and transcribed verbatim. Additionally, field notes and postscripts will be provided on the context and course of the interviews as well as the telephone conversations.

### Data analysis & Synthesis

Analysis of the data aims to provide case studies, which illustrate key issues within a process of care including perspectives of older people living alone and their non-kin-carers. Therefore, we refer to strategies applied in longitudinal case study research (e.g. [48, 49]) as well as the grounded theory approach. The latter is used when little is known about the area of interest and it focuses on identifying, describing and explaining interactional processes in a social context [50].

Analysis will be iterative throughout the study, which allows to condense emerging themes and phenomena within trajectories as well as to inform subsequent interviews [51]. We will apply MAXQDA software to manage qualitative data, specifically for coding as well as for comparison within and across cases and trajectories [52]. The aim is to create a narrative of the case, in order to work out key phenomena according to the research questions. In light of these, analysis should also result in a systematically characterization of different care arrangements which support older people’s preferences for staying at home.

In addition to the analysis of trajectories, we will conduct cross-sectional analysis for selected data-sets, e.g. baseline interviews with the older people living alone and the non-kin-carers as well as retrospective interviews with bereaved non-kin-carers [36]. To this end, the data will be coded using MAXQDA software and categories will be arranged in order to work out key phenomena and formulate theoretical assumptions following grounded theory methodology in a constructivist understanding as suggested by [53].

### Quality assurance

In qualitative research, and especially in end-of-life studies support for the researcher is essential in order to ensure high quality data and avoid distancing and/or over-involvement [54]. As this is a longitudinal study specific risks have to be considered, such as over-rapport [33] or concerns with regard to death and dying of participants. However, it is important to reflect upon these. Beyond ongoing supervision by the project leader throughout the study the research team will require counseling by an external supervisor (psychologist) to address burdensome experiences during the fieldwork.

#### Inter- & Transdisciplinary Reflective Workshops 

Through reflective workshops, we aim at theory building throughout the research process. Theoretical issues of this study, such as gender or quality of care in formal and informal settings, will be of interest, as well as ethical, methodological and practical aspects of conducting the research project. Investing in quality assurance of the research is a major concern.

In order to capture this, we plan up to three half-day workshops a year. The core team will reflect with the cooperation partners in up to two interdisciplinary workshops (int-WS) per year. This will be complemented/amended by annually transdisciplinary workshops (trans-WS), including representatives of non-academic stakeholder groups like NGO’s and informal carer interest groups.

A facilitator who is not a member of the research team but familiar with qualitative research in end-of-life care should guide these discussions. This has proven to be good practice in inter- and transdisciplinary research settings [55]. For each of these meetings, relevant material will be prepared beforehand by the core-team, and a written protocol will be made afterwards to note the key issues (see Table 3).

**Table 3:** Workshop schedule

Year One:	Reflection on process of recruitment and sampling, Specific analysis and comparison of literature on gender issues and formal-informal care encounters in home care, Developing interview guides (*two int-WS, one trans-WS*)
Year Two:	Reflection on the interview dynamics, Reflection on Analysis of baseline interviews (*one int-WS, one trans-WS*)
Year Three:	The process of developing case studies, Discussing case studies, Synthesis of results, Presenting data & Dissemination (*two int-WS, one trans-WS*)

### Project schedule

This research project will last from March 2018 to February 2021 (36 months).

#### Phase 1 - preparation (3–6 Mon)

This phase will consist of an update of the literature and the state of research. Further submission of application for ethical review at the relevant ethic committees is a key activity as well as providing field access and preparing fieldwork.

#### Phase 2 - data collection & fieldwork (20–24 Mon)

The conduct of baseline and serial interviews, regular telephone contacts as well as the retrospective interview are core activities. Each trajectory will be followed up to 18 months. Considering a consecutive start of data collection as well as a period of 3–5 months after death for a retrospective interview we calculate a period of 24 months for fieldwork.

#### Phase 3 - analysis (22 Mon)

Data analysis is an iterative process and will start parallel to data collection, however, intensity will vary over time, e.g. analysis of baseline interviews, development of case studies, etc.

#### Phase 4 - synthesis & discussion (6 Mon)

The last 6 months of this study will focus on systematically comparing results from analysis and discussion in light of the various theoretical discourses.

#### Phase 5 - dissemination (6 Mon+)

While a narrow focus on disseminating the results at different levels is put on in the last phase of the study, earlier dissemination will happen in the way of presentations given at conferences or papers written. This interdisciplinary study aims to address various scientific communities (e.g. nursing science, public health, gerontology, geriatrics, social sciences) as well as health care professionals and service providers including the wider public. We put a focus on Austria when approaching *health professionals, service providers and interest groups* with the conduct and results of this study. Apart from involving non-academic stakeholders in the planned annual transdisciplinary workshops, we will contribute with presentations at national conferences or meetings as well as project letters which will be disseminated through websites of the institutes involved as well as partners like the Austrian Interest group of informal caregivers.

## Discussion

Non-kin-care for older people living alone has to be considered underexplored, at least in the German speaking context, which has to do with the residual development of gerontology as well as nursing research or health care services research. However, including a focus on end-of-life and gender issues in this non-organized field of care is innovative even for the wider international scientific community. When looking at formal-informal care encounters the focus is either on family caregivers or on voluntary work (organized forms). Apart from this, the gender perspective, relevant in both, recipients and providers of care, will provide new insights into new ways of coping with challenges.

Widening the perspectives of care arrangements through going beyond family-relationships and reaching out to develop a culture of care in society is a major contribution we expect from this work, as non-kin-care arrangements are exemplary for civic involvement in care. To promote these forms of engagement we need basic research in this field to better understand motivation and needs of these carers. This allows new perspectives on collaboration between civic engagement and professional services in a welfare mix which extends traditional understandings of informal caregiving members [56].

This study protocol introduces longitudinal qualitative research methodology [57] in the field of home care, which is a rather young development in palliative care research and has not been conducted in Austria so far. Most research is limited to snapshots, but this approach allows investigating the procedural aspects of care, including decision-management in the trajectories towards the end of life. In addition, this study is interdisciplinary by nature, and the research team has different backgrounds, including gerontology, nursing and health care sciences, palliative care and health economics. Investing in additional reflexivity and communication procedures creates innovative results and robust knowledge as outcomes [55].

## Data Availability

Not applicable.

## Abbreviations

EC: Ethics Committee
FWF: Austrian Science Fund
GP: General Practitioner
OPLA: Older People Living Alone
RI: Retrospective Interview
WS: Workshop
